# Invasive Plants: Turning Enemies into Value

**DOI:** 10.3390/molecules25153529

**Published:** 2020-08-01

**Authors:** Patrícia Máximo, Luísa M. Ferreira, Paula S. Branco, Ana Lourenço

**Affiliations:** LAQV-REQUIMTE, Department of Chemistry, NOVA School of Science and Technology, NOVA University, 2829-516 Caparica, Lisbon, Portugal; lpf@fct.unl.pt (L.M.F.); paula.branco@fct.unl.pt (P.S.B.); ana.lourenco@fct.unl.pt (A.L.)

**Keywords:** *Carpobrotus edulis*, *Hakea salicifolia*, *Hakea sericea*, *Oxalis pes-caprae*, *Phytolacca americana*

## Abstract

In this review, a brief description of the invasive phenomena associated with plants and its consequences to the ecosystem is presented. Five worldwide invasive plants that are a threat to Portugal were selected as an example, and a brief description of each is presented. A full description of their secondary metabolites and biological activity is given, and a resume of the biological activity of extracts is also included. The chemical and pharmaceutical potential of invasive species sensu lato is thus acknowledged. With this paper, we hope to demonstrate that invasive species have potential positive attributes even though at the same time they might need to be controlled or eradicated. Positive attributes include chemical and pharmaceutical properties and developing these could help mitigate the costs of management and eradication.

## 1. Introduction

Invasive species are a menace to the ecosystem of their surroundings. These invasions are one of the great threats to biodiversity, since invasive species establish and supersede native species, frequently leading to the extinction of the latter. Invasive plant interactions in the ecosystem comprise the alteration of abiotic or biotic conditions such as nutrient and water availability, and the disturbance of bacterial and fungal communities, as well as of plant–plant and plant–herbivore interactions. Allelopathic compounds may be released by invaders and fire regimes are also affected, whilst the derived increase in decomposition of organic matter influences the nitrogen and carbon cycles. All of these influence the remaining organisms, micro-, animal or vegetable, thus compromising and altering the established biodiversity [1,2]. Apart from the ecological impact, they also have a socio-economic impact by influencing human-health, infrastructures and local economies [3,4,5]. Invasive species are presently one of the concerns of the European Union [6,7], and particularly of Portugal [8,9], where several species have been recognized.

The plant invasive phenomenon begins with their introduction (accidental or deliberate), proceeds by establishment of the species (through biotic and abiotic factors), and ends with its spread and impact [10]. Several theories of the mechanisms involved in the invasion process have been advanced (that may very well act together), including the enemy release hypothesis, the evolution of increased competitive ability, the novel weapon hypothesis and the allelopathic advantage against resident species hypothesis: in short, invasive plants face less or no enemies or predators in the new ecosystem, can thus redirect resources to favor establishment, being more competitive than local species, and may very well develop new biochemical weapons [3,10].

The management of the invasion problem includes many features like risk assessment, vector management, early detection, eradication, mitigation and restoration [5]. Of these, and for the perception of the general audience, early detection, sometimes with the help of the community, and mitigation are the most obvious, the latter being achieved by mechanical means, biological control, and/or chemical remedies. Interesting reviews on the control of invasive species and maintenance of biodiversity can be found in the literature [11,12]. Nevertheless, all management and control actions have costs that, in Europe, are estimated as millions of euros [13]. The search for alternative measures to the current status should thus be addressed. An alternative use for these species should be obtained, since eradication is far from being attained in most cases. We here suggest their use as a source of potential pharmaceuticals that, once available, could generate income, thus reducing the global costs of eradication. We must, however, be cautious in this approach—we do not want to sustain the targeted species, but rather eradicate them. Therefore, we propose eradication procedures that include not incinerating these species or burying them in landfills but rather processing them for chemical constituents. The delicate subject of harvest incentives is already the focus of an interesting review by Pasko and Goldberg [14]: attention must be paid to several points including: biological (population dynamics, overcompensation and dispersal), ecological, and socioeconomical (management goals, market economics) factors, among others. This type of approach would fall in the category of ‘commercial use’ whose issues and risks have also been the subject of the study of the French National Work Group on Biological Invasions in Aquatic Environments (IBMA) [15]. The temporary commercial use of established invasive species is already foreseen in European regulations (provided it is included in management measures aiming eradication) [16].

Furthermore, we do not intend to use invasive species in traditional medicines or phytotherapies. We defend the search for active principles and scaffolds, together with detailed pharmacological and toxicity studies, namely the usual route to the discovery of lead molecules. And for that, we must start at the beginning. First, we need to acknowledge the underlying potential of invasive species. Their ease of adaptation and control of the new habitat implies adaptation to different soil compositions, different water and weather stress conditions, ability to find reproductive strategies, competitive advantage, and, of course, resistance to new predators. For that, plants rely on their chemical machinery: their ability to synthesize allelopathic or deterrent compounds may very well mean the difference between life and death, especially when it comes to resistance to predators. The chemistry of invasive species must, as such, be very varied and with an enormous biological activity potential that remains yet to be explored. This, of course, implies new studies in this area, focusing on biological activity of isolated products—most of the existing phytochemical studies of invasive species focus on biological activity of extracts and no active principles were isolated; the search for the active metabolites should thus be a priority. It is also desirable that these studies focus on the species as invaders, and not on their native constitution. Most probably the prevalence of invasive species over endemic ones relies on a yet unknown biological activity of the metabolites they produce; these are surely responsible for their ease of expansion and dominance of the new habitat, and so their chemistry can surely be correlated to their ability to survive in non-native ecosystems. Moreover, the use of these species as a source of therapeutics would allow a rational use of resources that would eventually mitigate the cost of their removal. Are phytochemistry and bioactive natural products the miraculous solution to the invasive problem? Of course not, but they may be something worth trying, as we are trying to illustrate in this review.

In this paper, we chose worldwide invasive species that are a threat to Portugal where they are recognized by government [8] and the scientific community [9], in order to illustrate their potential as a source of bioactive metabolites: *Carpobrotus edulis*, *Hakea salicifolia*, *Hakea sericea, Oxalis pes-caprae* and *Phytolacca americana*. They are all invasive to Mainland Portugal, and *Carpobrotus edulis*, *Oxalis pes-caprae* and *Phytolacca americana* are also invasive to Madeira and Azores. Some of them are recognized by the European and Mediterranean Plant Protection Organization either as Pests (*Hakea sericea*, invasive also to Spain and France) or Invasive Plants (*Carpobrotus edulis*, invasive also to Spain, France, UK, Italy, Malta and Israel, and *Oxalis pes-caprae*, invasive also to Malta, Georgia, and Israel). Although their chemistry is poorly studied, either as native or invasive, several reports exist on the biological activity of their extracts that could, and should, be further explored.

## 2. *Carpobrotus edulis* L.

*Carpobrotus edulis* (common name ice plant, Aizoaceae) is a succulent perennial subshrub that can grow to several meters tall. It was introduced in Portugal for ornamental purposes where it is grown for maintenance of dunes and slopes. It shows vigorous growth leading to the formation of continuous vegetative areas that prevent the existence of native vegetation. It promotes soil acidification and can exist in damp or dry areas. It is native to South Africa [9], where it finds use in traditional medicine for symptoms of tuberculosis, throat infections, diarrhea, dysentery, burns, stomach ailments, chilblains, mouth ulcers, sinusitis and diabetes [17]. It is also invasive in Southern Europe, Western USA, New Zealand and North Africa [18]

**Figure 1:**
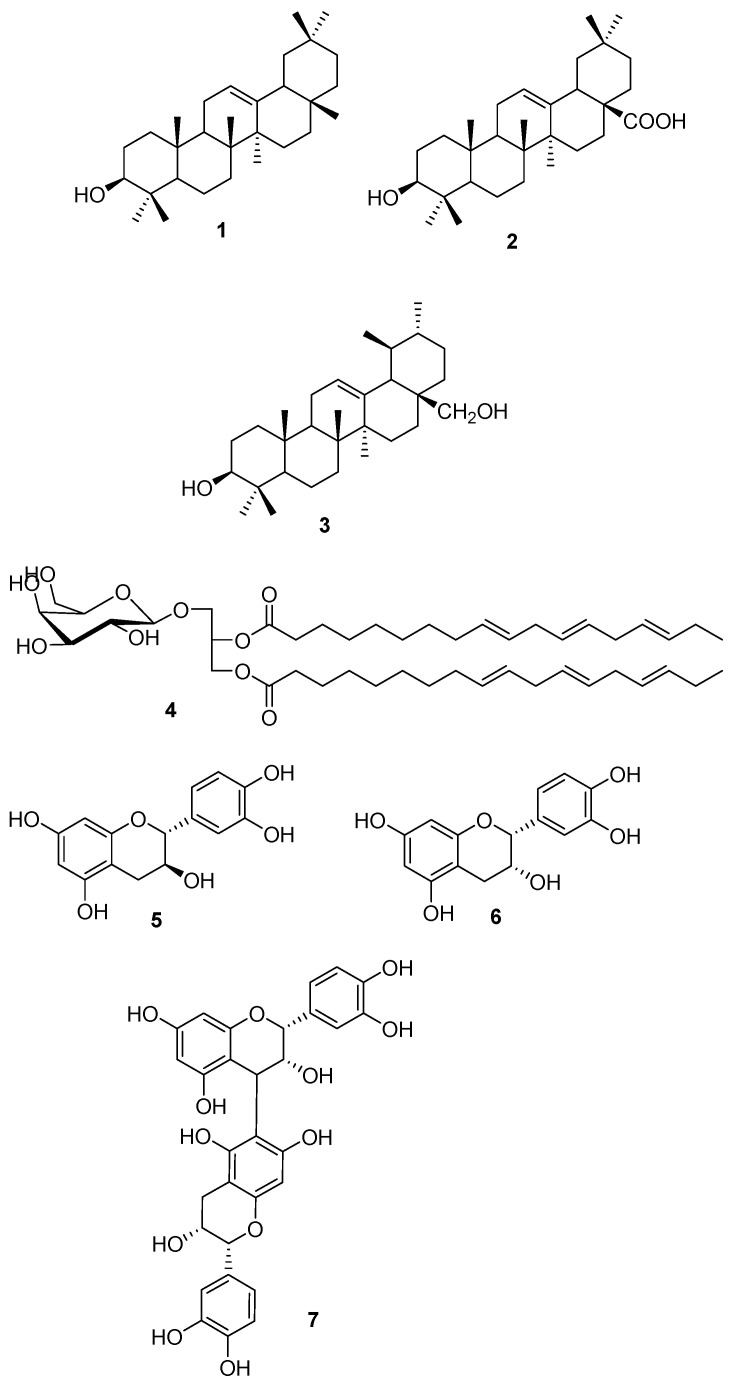
Compounds isolated from *C. edulis* [19] β-amyrin **1**, oleanolic acid **2**, uvaol **3**, monogalactosyldiacylglycerol **4**, catechin **5**, epicatechin **6** and procyanidin B5 **7**.

From a study of the MeOH extract of a population collected in Sintra, Portugal, the compounds in Figure 1 were isolated [19].

Their ability to inhibit P-glycoprotein (the efflux pump responsible for the multidrug resistance of the used cell line) of mouse lymphoma cells containing the human efflux pump gene MDR1 and their antibacterial activity was studied [19,20]: uvaol **3** was the most effective and promising compound in the reversal of multidrug resistance in MDR mouse lymphoma cell line, whilst oleanolic acid **2** presented high antibacterial activity against a large number of bacterial strains [20].

There have been several studies on the biological activity of extracts of this species (Table 1). We can find Anti-Proteus [21] and anti-Klebsiella [22] activities of the MeOH and water extracts of South African species, indicating their potential for blocking the onset of rheumatoid arthritis and preventing the onset of ankylosing spondylitis [21,22]; Inhibition on the growth of phagocytosed multidrug-resistant *Mycobacterium tuberculosis* and methicillin-resistant *Staphylococcus aureus* of the MeOH/water extract of a species from Sintra, Portugal, suggesting that this plant may serve as a source of new antimicrobial agents that are effective against problematic drug-resistant intracellular infections [23]; neuroprotective properties of the *n*-hexane, CH_2_Cl_2_, AcOEt and MeOH extracts of a species from Faro, Portugal, suggesting that the consumption of leaves from *C. edulis* can contribute for a balanced diet and may add to the improvement of cognitive functions [24]; the effect of the MeOH/water extract of an undisclosed species in inhibiting the MDR efflux pumps, enhancing the killing of phagocytosed *S. aureus* and promoting immune modulation, indicating that the resistance modifier and immunomodulatory effect of this plant extract can be exploited in the experimental chemotherapy of cancer and bacterial or viral infections [25]; antioxidant, metal chelating and anticholinesterase activities of MeOH extracts of species collected in the Algarve, Portugal, together with their fatty acid profile, indicating that *C. edulis* is a candidate on novel and alternative therapies for the treatment of neurological disorders associated with low levels of acetylcholine in the brain [26]; antioxidant and antimicrobial activity of the acetone/water extract of a species collected in Monastir, Tunisia emphasizing the beneficial cosmetic and therapeutic use of this plant [27]; antioxidant activity of the *n*-hexane, acetone, EtOH and water extracts of a species from Eastern Cape, South Africa, that may justify the traditional use of this plant in the management of common diseases in HIV/AIDS patients in Eastern Cape Province [28], and inhibition of protein glycation, antioxidant and antiproliferative activities of the EtOH and EtOH/Water extracts of species collected in Sousse, Tunisia [17]. From this study, and by HPLC analysis with standards, sinapic acid, ferulic acid, luteolin 7-*O*-glucoside, hyperoside, isoquercitrin, ellagic acid and isorhamnetin 3-*O*-rutinoside were identified [17]. The results suggest that the *C. edulis* extracts could be used as an easily accessible source of natural antioxidants and as potential phytochemicals against protein glycation and colon cancer. More recently a study concerning the biological activity of the peel and flesh extracts (water, EtOH and acetone) of the fruits of a specimen of *C. edulis*, collected in Algarve, Portugal, was published [29]. Antioxidant, anti-microbial, enzymatic inhibitory properties and toxicity were evaluated and more than 80 compounds (mostly phenolic acids, flavonoids, and coumarins) were identified by HPLC-ESI-MS/MS, with or without standards. The potential use of the fruits of *C. edulis* as sources of molecules and/or products to be used in the food, pharmaceutic, agriculture and cosmetic areas is suggested.

## 3. *Hakea salicifolia* (Vent.) B. L. Burtt and *Hakea sericea* Schrader

*Hakea salicifolia* (common name willow-leaved Hakea, Proteaceae) is a perennial shrub or small tree (up to 5 m) with reddish twigs. It was introduced in Portugal for ornamental purposes and for the formation of hedges in windy zones, near the shore. It is well adjusted to nutrient depleted soils, preferring sunny areas. It is native to Southeast Australia and Tasmania [9]. It is also invasive in Europe, Australia, New Zealand and South Africa [18]

*Hakea sericea* (common name silky Hakea, Proteaceae) is a perennial shrub or small tree (up to 4 m) with irregular top and robust and very sharp needle-like leaves. It was introduced in Portugal for ornamental purposes and for the formation of hedges. It prefers disturbed areas such as along the sides of roads. It is resistant to wind and drought. It is native to Southern Australia [9]. It is also invasive in Southern Europe, New Zealand and South Africa [18].

**Figure 2:**
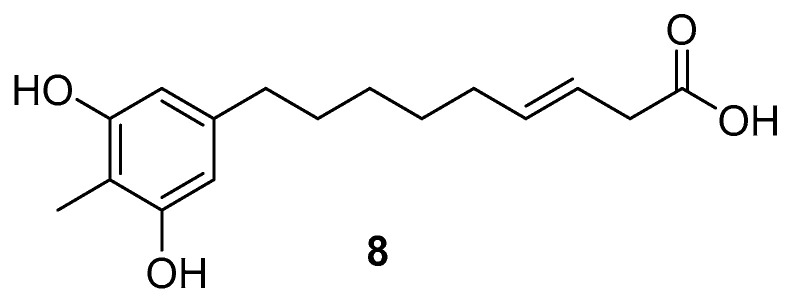
9-(3,5-dihydroxy-4-methylphenyl)nona-3(*Z*)-enoic acid **8** from *H. sericeae* [30,31].

Chemical studies on this species refer only to the isolation of 9-(3,5-dihydroxy-4-methylphenyl)nona-3(*Z*)-enoic acid **8** (Figure 2) from the MeOH extract of the fruits of *H. sericeae* collected in Serra da Estrela, Portugal [30,31].

The antibacterial properties of this new alkenylresorcinol were studied against several strains of Gram-positive and Gram-negative bacteria using the resazurin microtiter assay. Good MIC values were obtained against *Staphylococcus aureus* strains (0.005–0.16 mg/mL), including the clinical isolates (SA 01/10, SA 02/10 and SA 03/10) and MRSA strains [30]. The possible economical valorization of this species is suggested, based on the putative use of this compound in the preservation of foods or as an alternative to conventional antibiotic therapy [31].

Three reports can be found on the biological activity of extracts of these species (Table 2). These comprise the antimicrobial activity of *n*-hexane, CH_2_Cl_2_, EtOAc, MeOH and water extracts of both species, collected at Lisbon, Portugal, against Gram-positive and Gram-negative bacteria, including *S. aureus* MR where the twigs’ aqueous extract showed the strongest antimicrobial activity (MIC 7.5–62 μg/mL) against the tested methicillin and vancomycin resistant strains of *S. aureus* [32]; the antioxidant potential of MeOH extracts of *H. sericeae*, collected at Serra da Estrela, Portugal [33], and the antimicrobial, antibiofilm and cytotoxic activities of the MeOH extracts of *H. sericea* collected at Serra da Estrela, Portugal, demonstrating that *H. sericea* is a potential source of bioactive compounds with antimicrobial activity, namely against several *S. aureus* strains, including clinical MRSA [34].

## 4. *Oxalis pes-caprae* L.

*Oxalis pes-caprae* (common name bermuda buttercup, Oxalidaceae) is a perennial herb (up to 40 cm) with bulbills. It was probably introduced for ornamental purposes. It grows in cultivated lands and bare places, especially on loamy soils. It does not stand the frost and low temperature that lead to dryness of the aerial parts. It is native to South Africa (Cape region) [9]. It is also invasive in Mediterranean Europe, Western USA, Asia, Australia, New Zealand and South Africa [18].

Oxalis species owe their sour taste to the presence of oxalic acid, a toxic compound that may cause nervous system paralysis in large herbivores when consumed in great quantities [35]. Several Oxalis species have been used in folk medicine due to their antihypertensive effects [35].

**Figure 3:**
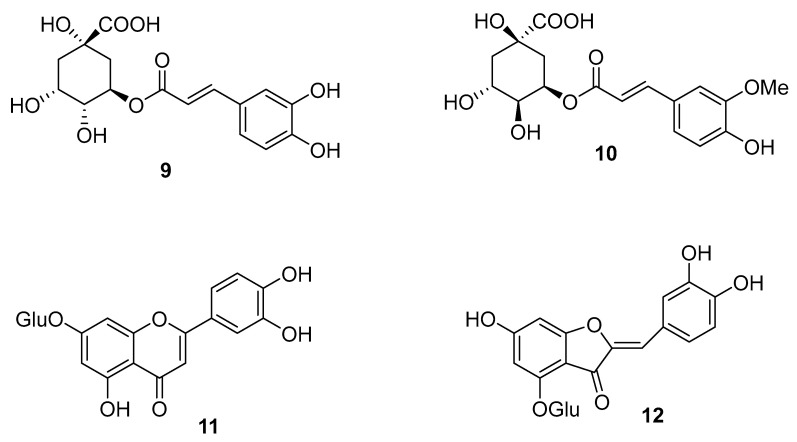
Phenolics identified from *Oxalis pes-caprae* [36]. Chlorogenic acid **9**, quinic ferrulate **10**, luteolin glucoside **11** and cernuoside **12**.

Few reports exist on the chemistry of this species. These include the identification of phenolics and flavonoids from the EtOAc, MeOH and BuOH/water extracts of a specimen collected in Crete (Figure 3) [36]. While compound **12** was isolated, compounds **9–11** were tentatively identified by LC-DAD-MS. The extracts exhibited high levels of anti-oxidant activity and the authors suggest that these invasive plants may serve as an inexpensive and renewable source of bioactive compounds [36].

**Figure 4:**
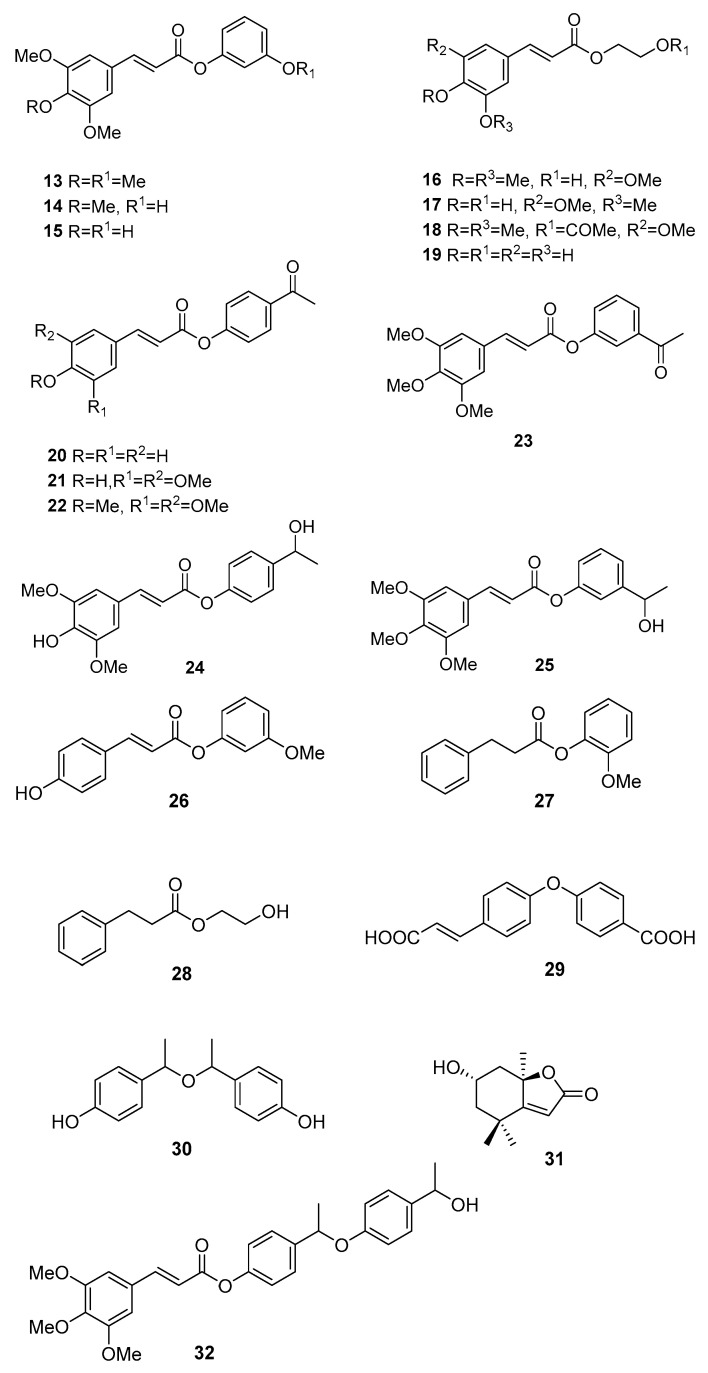
Compounds from *Oxalis pes-caprae* [37,38,39,40]. (*E*)-3-methoxyphenyl 3,4,5-trimethoxycinnamate **13**, (*E*)-3-hydroxyphenyl 3,4,5-trimethoxycinnamate **14**, 3-hydroxyphenyl sinapate **15**, (*E*)-2-hydroxyethyl 3,4,5-trimethoxycinnamate **16**, 2-hydroxyethyl sinapate **17**, (*E*)-2-acetylethyl 3,4,5-trimethoxycinnamate **18**, 2-hydroxyethyl caffeate **19**, 4′-acetylphenyl 4-hydroxycinnamate **20**, 4′-acetylphenyl 4-hydroxycinnamate **21**, 4′-acetylphenyl 4-*O*-methylsinapate **22**, 3′-acetylphenyl 4-methylsinapate **23**, 4′-(1-hydroxyethyl)phenyl sinapate **24**, 3′-(1-hydroxyethyl)phenyl 4-methylsinapate **25**, (*E*)-3-methoxyphenyl 4-hydroxycinnamate **26**, 2-methoxyphenyl 3-phenylpropanoate **27**, 2-hydroxyethyl 3-phenylpropanoate **28**, (*E*)-4-[4-(2-carboxyethenyl)phenoxy]benzoic acid **29**, 4,4′-[1,1′-oxybis(ethane-1,1-diyl)]diphenol **30**, loliolide **31**, and (E)-4-(1-(4-(1-hydroxyethyl)phenoxy)ethyl)phenyl 3,4,5-trimethoxycinnamate **32**.

Studies of DellaGreca et al. on the AcOEt, MeOH and water extracts of specimens collected in Bacoli, Naples, where this species in invasive on cultivated lands, led to the isolation of the compounds in Figure 4, together with common phenolics [37,38,39,40]. These include *p*-coumaric acid, dihydrocinnamic acid, cis-*p*-coumaric acid, cinnamic acid, 1,2,3,4-tetrahydro-1-methyl-β-carboline-3-carboxylic acid, 3-methoxyphenol, 2-methoxyphenol, 4-hydroxybenzoic acid, 4-(1-hydroxyethyl)phenol, and 3-(1-hydroxyethyl)phenol. The isolated compounds were tested as to their activity towards the germination and growth of Lactuca sativa (lettuce). The phytotoxicity observed for some of these compounds on germination and growth of lettuce seeds seems to contribute to the invasiveness of the plant and their use as agrochemicals if suitably prepared and/or modified is suggested [37,38,39].

Further reports include the studies of an extract of the leaves of an undisclosed specimen towards vascular, antioxidant and neuroprotective activities, suggesting the potential use of this extract as a source of bioactive compounds [35].

## 5. *Phytolacca americana* L.

*Phytolacca americana* (common name pokeweed, Phytolaccaceae) is a big branched herb (up to 3 m) sometimes lignified at the base. It was introduced for medicinal purposes and for use in dyeing. It exists in disturbed and ruderal habitats, agricultural fields and along the sides of roads. It is native to North America [9]. It is also invasive in Europe and Western USA [18].

**Figure 5:**
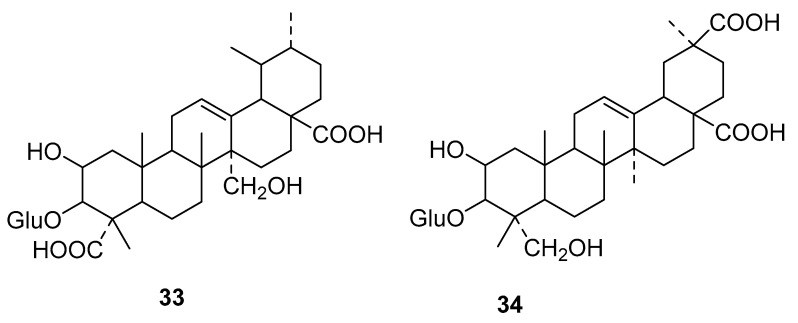
Compounds identified by LC/MS on a Chinese specimen of *Phytolacca americana* [41]. Esculentoside isomer **33** and esculentoside P **34**.

On the chemistry of this species we can find the isolation of saponins in the works of Ding et al. on acaricidal activity of the petroleum ether, acetone and MeOH extracts of a Chinese specimen. By LC/MS the two compounds in Figure 5 were identified [41].

Among the *P. americana* extracts evaluated, the root acetone extract showed the highest acaricidal activity for *T. cinnabarinus* female adults [41].

The work of Jeong et al. reports the isolation of α-spinosterol from the MeOH extract of the roots of a Korean specimen and its action on diabetic Nephropathy, suggesting that this compound has a significant therapeutic potential [42], while Jerz et al. report the isolation of betalains from the berries of an undisclosed specimen [43].

**Figure 6:**
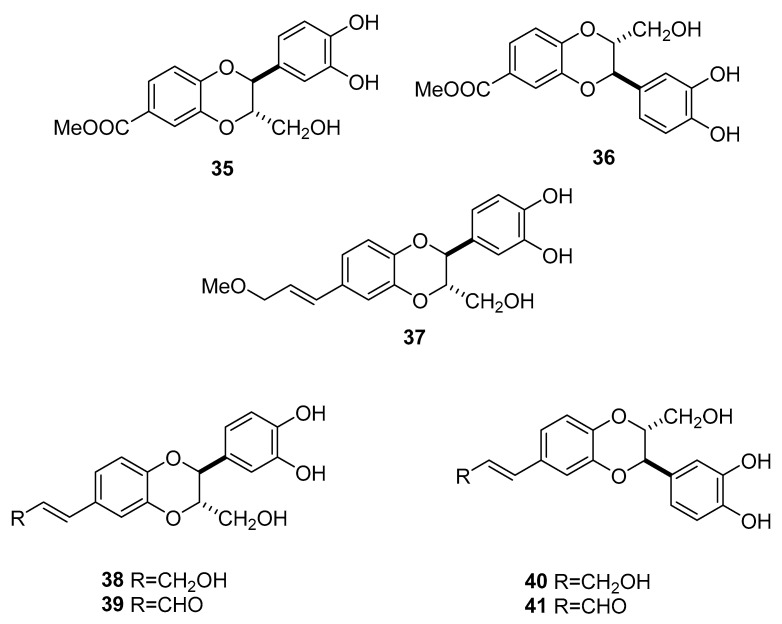
1,4-benzodioxane derivatives of *Phytolacca americana* [46]. Americanoic acid methyl ester **35**, isoamericanoic acid A methyl ester **36**, 9′-*O*-methylamericanol A **37**, americanol A **38**, americanin A **39**, isoamericanol A **40**, and isoamericanin A **41**.

Works of Takahasi et al. report the isolation of 1,4-benzodioxane derivatives from the MeOH extract of the seeds of Japanese specimens and their neuritogenic activity in primary cultured rat cortical neurons, suggesting their role as potential candidates for nonpeptide neurotrophic agents (Figure 6) [44,45,46]. The saponins esculentoside B and esculentside S were also isolated [46].

Reports on the biological activity of extracts of this species include (Table 3): moluscidal activity of the water extract of the berries against invasive snails (*Viviparus georgianis* and *Pimephales promelas*) suggesting that *P. americana* could be used as a mollusk control agent in aquaculture applications [47]; antifungal activity of the MeOH/water extracts of the aerial parts of a Korean species towards phytopathogenic fungi, confirming that extracts originated from invasive plants can be used directly to develop new and effective classes of natural fungicides to control severe fungal diseases [48]; allelopathic activity of the aqueous leaf extract of a South Korean species on *Cassia mimosoides* [49]; antibacterial effect of MeOH/water extract of aerial parts of a Korean species on pathogens responsible for periodontal inflammatory diseases and dental caries, suggesting that these extracts have the potential for use in the preparation of toothpaste and other drugs related to various oral diseases [50]; antiproliferative activity of the EtOH/water extract (saponin rich) of the roots of a Chinese specimen [51] and inhibition of infection by Cucumber Mosaic virus and Influenza virus by a phosphate buffer extract of the leaves of a California specimen [52].

Finally, a patent registers a method for treating all types of polycystic kidney disease using the herb *Phytolacca americana*, among others [53].

## 6. Conclusions

In this review, we chose examples of shrubs (*Hakea*), herbs (*Oxalis* and *Phytolacca*) and a succulent plant (*Carpobrotus*) to illustrate the varied chemical and pharmaceutical potential of invasive plants. Although poorly studied from the perspective of beneficial attributes, as most invasive species are, the extracts of these species show interesting biological activities, ranging from antioxidant, antimicrobial and antifungal, to neuroprotective and neuritogenic, including antiproliferative and cytotoxic, anticholinesterase, allelopatic and inhibition of viral growth. We thus clearly demonstrate the chemical potential of several kinds of invasive species, potential that should be further explored—invasive plants pose an up to date problem that should be turned into a profitable resource. The use of invasive species as a source of active metabolites could help reduce the actual and future costs of control and management, becoming that added value resource. As such, additional efforts should be directed towards the phytochemical study of these species in their invasive habitat. These studies should be complemented with a large scope analysis of bioactivities of isolated products, such as antimicrobial, antioxidant, anticancer/antiproliferative, and anti-inflammatory activities, among others. This of course is only the beginning—time will tell if there is in fact any use for the isolated bioactive metabolites: the discovery of pharmaceutical lead compounds is long, and substantial toxicity studies will also have to be made. We hope, however, to encourage the development of chemical studies of invasive species in the EU and worldwide, since they are most probably a source of active metabolites, and possibly of new active principle scaffolds. As such, we want to stimulate the scientific community to proceed with the thorough and detailed chemical analysis of invasive species at the same time eradication measures are being maintained. We want to alert the scientific community to the possibility of taking advantage of the metabolites produced by invasive species while eradicating them. We have no intention of valuing these species in order to delay or discourage their eradication, but rather to conduct studies on chemical composition and pharmacological application at the same time as control actions are being maintained.

## Figures and Tables

**Table 1:** Biological activity of extracts of *Carpobrotus edulis.*

Part of Plant	Extract	Activity	Refs.
undisclosed	MeOH and water	Anti-Proteus and anti-Klebsiella	[21,22]
leaves	MeOH/water	multidrug-resistant *Mycobacterium tuberculosis* and methicillin-resistant *Staphylococcus aureus*	[23]
leaves	*n*-hexane, CH_2_Cl_2_, AcOEt and MeOH	neuroprotective	[24]
leaves	MeOH/water	MDR efflux pumps, enhancing killing of phagocytosed *S. aureus* and promoting immune modulation	[25]
undisclosed	MeOH	antioxidant, metal chelating and anticholinesterase	[26]
leaves	Acetone/water	antioxidant and antimicrobial	[27]
leaves	*n*-hexane, acetone, EtOH and water	antioxidant	[28]
Undisclosed ^1^	EtOH and EtOH/water	inhibition of protein glycation, antioxidant and antiproliferative	[17]
Fruits ^2^	water, EtOH and acetone	Antioxidant, anti-microbial, enzymatic inhibitory activity	[29]

^1^ sinapic acid, ferulic acid, luteolin 7-*O*-glucoside, hyperoside, isoquercitrin, ellagic acid and isorhamnetin 3-*O*-rutinoside were identified; ^2^ more than 80 compounds (mostly phenolic acids, flavonoids, and coumarins) were identified.

**Table 2:** Biological activity of extracts of *Hakea* species.

Part of Plant	Extract	Activity	Ref.
leaves, twigs and fruits of *H. salicifolia* and leaves of *H. sericeae*	*n*-hexane, CH_2_Cl_2_, EtOAc, MeOH and water	Gram-positive and Gram-negative bacteria	[32]
Stems, leaves and fruits of *H. sericeae*	MeOH	antioxidant	[33]
Fruits of *H. sericea*	MeOH	antimicrobial, antibiofilm and cytotoxic	[34]

All these reports refer to *Hakea* species as invasive.

**Table 3:** Biological activity of extracts of *Phytolacca americana.*

Part of Plant	Extract	Activity	Ref.
Berries	water	*Viviparus georgianis* and *Pimephales promelas*	[47]
Aerial parts	MeOH/water	Phytopatogenic fungi	[48]
leaves	water	*Cassia mimosoides*	[49]
Aerial parts	MeOH/water	Antibacterial (periodontal inflammatory diseases and dental caries)	[50]
roots	EtOH/water	antiproliferative	[51]
leaves	phosphate buffer	Cucumber Mosaic and Influenza virus	[52]

## References

[1] Skurski T.C., Rew L.J., Maxwell B.D. (2014). Mechanisms underlying nonindigenous plant impacts: A review of recent experimental research. Invasive Plant. Sci. Manag..

[2] Zhang P., Li B., Wu J., Hu S. (2018). Invasive plants differentially affect soil biota through litter and rhizosphere pathways: A meta-analysis. Ecol. Lett..

[3] Sharma G.P., Singh J.S., Raghubanshi A.S. (2005). Plant invasions: Emerging trends and future implications. Curr. Sci..

[4] Shackleton R.T., Shackleton C.M., Kull C.A. (2019). The role of invasive alien species in shaping local livelihoods and human well-being: A review. J. Environ. Manag..

[5] Pyšek P., Richardson D.M. (2010). Invasive species, environmental change and management, and health. Annu. Rev. Environ. Resour..

[6] Beninde J., Fischer M.L., Hochkirch A., Zink A. (2015). Ambitious advances of the european union in the legislation of invasive alien species. Conserv. Lett..

[7] (2017). Invasive Alien Species of Union Concern.

[8] Decreto-Lei 565/99, de 21 de Dezembro do Ministério do Ambiente. Diário da República nº 295/1999, Série I-A de 21/12/1999. https://data.dre.pt/eli/dec-lei/565/1999/12/21/p/dre/pt/html.

[9] Marchante H., Morais M., Freitas H., Marchante E. (2014). Guia Prático Para a Identificação de Plantas Invasoras em Portugal.

[10] Rai P.K. (2015). What makes the plant invasion possible? Paradigm of invasion mechanisms, theories and attributes. Environ. Skept. Crit..

[11] Downey P.O., Williams M.C., Whiffen L.K., Auld B.A., Hamilton M.A., Burley A.L., Turner P.J. (2010). Managing alien plants for biodiversity outcomes—The need for triage. Invasive Plant. Sci. Manag..

[12] Weidlich E.W.A., Flórido F.G., Sorrini T.B., Brancalion P.H.S., Peralta G. (2020). Controlling invasive plant species in ecological restoration: A global review. J. Appl. Ecol..

[13] Brink P.T. The Economic Costs of Invasive Alien Species (IAS). https://www.iucn.org/sites/dev/files/import/downloads/ten_brink_economic_impacts_of_ias__ptb_of_ieep_at_the_iucn_ep_event_21_feb_2013_final.pdf.

[14] Pasko S., Goldberg J. (2014). Review of harvest incentives to control invasive species. Manag. Biol. Invasions.

[15] (2019). Making Use of Invasive Alien Species Settled in Natural Environments: An Effective Approach to Management?. http://especes-exotiques-envahissantes.fr/wp-content/uploads/2019/07/uicn-guide-eee-ang-m3.pdf.

[16] Regulation (EU) no 1143/2014 of the European Parliament and of the Council on the Prevention and Management of the Introduction and Spread of Invasive Alien Species. https://eur-lex.europa.eu/legal-content/EN/TXT/?uri=CELEX%3A32014R1143.

[17] Hafsa J., Hammi K.M., Ben Khedher M.R., Smach M.A., Charfeddine B., Limem K., Majdoub H. (2016). Inhibition of protein glycation, antioxidant and antiproliferative activities of carpobrotus edulis extracts. Biomed. Pharmacother..

[18] Invasive Plants in Portugal. http://invasoras.pt/en/species-fact-sheets/.

[19] Martins A., Vasas A., Schelz Z.S., Viveiros M., Molnar J., Hohmann J., Amaral L. (2010). Constituents of carpobrotus edulis inhibit p-glycoprotein of mdr1-transfected mouse lymphoma cells. Anticancer Res..

[20] Martins A., Vasas A., Viveiros M., Molnar J., Hohmann J., Amaral L. (2011). Antibacterial properties of compounds isolated from carpobrotus edulis. Int. J. Antimicrob. Agents.

[21] Cock I.E., Van Vuuren S.F. (2014). Anti-proteus activity of some south african medicinal plants: Their potential for the prevention of rheumatoid arthritis. Inflammopharmacology.

[22] Cock I.E., Van Vuuren S.F. (2015). The potential of selected south african plants with anti-klebsiella activity for the treatment and prevention of ankylosing spondylitis. Inflammopharmacology.

[23] Martins M., Ordway D., Kristiansen M., Viveiros M., Leandro C., Molnar J., Amaral L. (2005). Inhibition of the carpobrotus edulis methanol extract on the growth of phagocytosed multidrug-resistant mycobacterium tuberculosis and methicillin-resistant staphylococcus aureus. Fitoterapia.

[24] Rocha M.I., Rodrigues M.J., Pereira C., Pereira H., Da Silva M.M., Neng N.D., Nogueira J.M.F., Varela J., Barreira L., Custodio L. (2017). Biochemical profile and in vitro neuroprotective properties of *Carpobrotus edulis* L., a medicinal and edible halophyte native to the coast of south Africa. S. Afr. J. Bot..

[25] Ordway D., Hohmann J., Viveiros M., Viveiros A., Molnar J., Leandro C., Arroz M.J., Gracio M.A., Amaral L. (2003). Carpobrotus edulis methanol extract inhibits the mdr efflux pumps, enhances killing of phagocytosed s-aureus and promotes immune modulation. Phytother. Res..

[26] Custodio L., Ferreira A.C., Pereira H., Silvestre L., Vizetto-Duarte C., Barreira L., Rauter A.P., Albericio F., Varela J. (2012). The marine halophytes *Carpobrotus edulis* L. And *Arthrocnemum macrostachyum* L. Are potential sources of nutritionally important pufas and metabolites with antioxidant, metal chelating and anticholinesterase inhibitory activities. Bot. Mar..

[27] Meddeb E., Charni M., Ghazouani T., Cozzolino A., Fratianni F., Raboudi F., Nazzaro F., Fattouch S. (2017). Biochemical and molecular study of carpobrotus edulis bioactive properties and their effects on dugesia sicula (turbellaria, tricladida) regeneration. Appl. Biochem. Biotechnol..

[28] Omoruyi B.E., Bradley G., Afolayan A.J. (2012). Antioxidant and phytochemical properties of *Carpobrotus edulis* (L.) bolus leaf used for the management of common infections in hiv/aids patients in eastern cape province. BMC Complement. Altern. Med..

[29] Castañeda-Loaiza V., Placines C., Rodrigues M.J., Pereira C., Zengin Z., Uysal A., Jeko J., Cziáky Z., Reis C.P., Gaspar M.M. (2020). If you cannot beat them, join them: Exploring the fruits of the invasive species *Carpobrotus edulis* (L.) n.E. Br as a source of bioactive products. Ind. Crop. Prod..

[30] Luis A., Cruz C., Duarte A.P., Domingues F. (2013). An alkenylresorcinol derivative from hakea sericea fruits and their antimicrobial activity. Nat. Prod. Commun..

[31] Cruz C.P.A.F.M., Luís Â.F.S., Duarte A.P.C., Domingues F.D.C. (2013). Isolamento e identificação de um novo derivado de resorcinol, com atividade antimicrobiana, a partir dos frutos da planta hakea sericea shrader. PT Patent.

[32] Madureira A.M., Duarte A., Teixeira G. (2012). Antimicrobial activity of selected extracts from hakea salicifolia and h. Sericeae (proteaceae) against staphylococcus aureus multiresistant strains. S. Afr. J. Bot..

[33] Luis A., Domingues F., Duarte A.P. (2011). Bioactive compounds, rp-hplc analysis of phenolics, and antioxidant activity of some portuguese shrub species extracts. Nat. Prod. Commun..

[34] Luis A., Breitenfeld L., Ferreira S., Duarte A.P., Domingues F. (2014). Antimicrobial, antibiofilm and cytotoxic activities of hakea sericea schrader extracts. Pharm. Mag..

[35] Gaspar M.C., Fonseca D.A., Antunes M.J., Frigerio C., Gomes N.G.M., Vieira M., Santos A.E., Cruz M.T., Cotrim M.D., Campos M.G. (2018). Polyphenolic characterisation and bioactivity of an *Oxalis pes-caprae* L. Leaf extract. Nat. Prod. Res..

[36] Gucluturk I., Detsi A., Weiss E.K., Ioannou E., Roussis V., Kefalas P. (2012). Evaluation of anti-oxidant activity and identification of major polyphenolics of the invasive weed *Oxalis pes-caprae*. Phytochem. Anal..

[37] Dellagreca M., Previtera L., Purcaro R., Zarrelli A. (2007). Cinnamic ester derivatives from *Oxalis pes-caprae* (*bermuda buttercup*). J. Nat. Prod..

[38] Dellagreca M., Purcaro R., Previtera L., Zarrelli A. (2008). Phenyl cinnamate derivatives from oxalis pes-caprae. Chem. Biodivers..

[39] Dellagreca M., Previtera L., Purcaro R., Zarrelli A. (2009). Phytotoxic aromatic constituents of *Oxalis pes-caprae*. Chem. Biodivers..

[40] Dellagreca M., Previtera L., Zarrelli A. (2010). A new aromatic component from *Oxalis pes-caprae*. Nat. Prod. Res..

[41] Ding L.J., Ding W., Zhang Y.Q., Luo J.X. (2013). Bioguided fractionation and isolation of esculentoside p from *Phytolacca americana* L.. Ind. Crop. Prod..

[42] Jeong S.I., Kim K.J., Choi M.K., Keum K.S., Lee S., Ahn S.H., Back S.H., Song J.H., Ju Y.S., Choi B.K. (2004). Alpha-spinasterol isolated from the root of phytolacca americana and its pharmacological property on diabetic nephropathy. Planta Med..

[43] Jerz G., Skotzki T., Fiege K., Winterhalter P., Wybraniec S. (2008). Separation of betalains from berries of phytolacca americana by ion-pair high-speed counter-current chromatography. J. Chromatogr. A.

[44] Hasegawa T., Fukuyama Y., Koshino K., Nakagawa K., Tori M., Asakawa Y. (1987). Structure of isoamericanin a, a prostaglandin 1 2 inducer, isolated from the seeds of *Phytolacca americana* L.. Chem. Lett..

[45] Fukuyama Y., Hasegawa T., Toda M., Kodama M., Okazaki H. (1992). Structures of americanol a and isoamericanol a having a neurotrophic property from the seeds of phytolacca americana. Chem. Pharm. Bull..

[46] Takahasi H., Yanagi K., Ueda M., Nakade K., Fukuyama Y. (2003). Structures of 1,4-benzodioxane derivatives from the seeds of phytolacca americana and their neuritogenic activity in primary cultured rat cortical neurons. Chem. Pharm. Bull..

[47] Aldea M., Allen-Gil S. (2005). Comparative toxicity of pokeweed (*phytolacca* americana) extracts to invasive snails (*viviparus* georgianis) and fathead minnows (*pimephales* promelas) and the implications for aquaculture. Bull. Environ. Contam. Toxicol..

[48] Bajpai V.K., Baek K.H., Kim E.S., Han J.E., Kwak M., Oh K., Kim J.C., Kim S., Choi G.J. (2012). In vivo antifungal activities of the methanol extracts of invasive plant species against plant pathogenic fungi. Plant Pathol. J..

[49] Kim Y.O., Johnson J.D., Lee E.J. (2005). Phytotoxicity of phytolacca americana leaf extracts on the growth, and physiological response of cassia mimosoides. J. Chem. Ecol..

[50] Patra J.K., Kim E.S., Oh K., Kim H.J., Kim Y., Baek K.H. (2014). Antibacterial effect of crude extract and metabolites of phytolacca americana on pathogens responsible for periodontal inflammatory diseases and dental caries. BMC Complement. Altern. Med..

[51] Saleri F.D., Chen G.L., Li X., Guo M.Q. (2017). Comparative analysis of saponins from different phytolaccaceae species and their antiproliferative activities. Molecules.

[52] Tomlinson J.A., Walker V.M., Flewett T.H., Barclay G.R. (1974). Inhibition of infection by cucumber mosaic-virus and influenza-virus by extracts from phytolacca-americana. J. Gen. Virol..

[53] Yarnell E. (2019). Herbal Medicines for Treating Polycystic Kidney Diseases. International Application.

